# Impact of sequential pediatric pneumococcal conjugate vaccination (PCV10/PCV13/PCV10) on serotype distribution and antimicrobial resistance in invasive and non-invasive *Streptococcus pneumoniae* isolates in Serbian children

**DOI:** 10.3389/fmicb.2026.1801133

**Published:** 2026-04-21

**Authors:** Milos Jovicevic, Jovana Kabic, Dusan Kekic, Vesna Kovacevic-Jovanovic, Zorica Vasiljevic, Olivera Hadzi-Simovic, Aleksandra Vukicevic Lazic, Suzana Laban-Nestorovic, Tamara Djordjevic, Anita Sente Zigmanovic, Tatjana Stojsic, Aleksandar Ilic, Lazar Ranin, Ina Gajic, Natasa Opavski

**Affiliations:** 1 Institute of Microbiology and Immunology, Faculty of Medicine, University of Belgrade, Belgrade, Serbia; 2 Medigroup Health System, Central Laboratory, Belgrade, Serbia; 3 Department of Clinical Microbiology, Mother and Child Health Care Institute of Serbia "Dr.Vukan Cupic”, Belgrade, Serbia; 4 Department of Microbiology, University Children's Hospital, Belgrade, Serbia; 5 General Hospital Subotica, Subotica, Serbia; 6 General Hospital “Dr Laza K. Lazarevic”, Sabac, Serbia; 7 Clinical Hospital Centre Zemun, Belgrade, Serbia

**Keywords:** IPD, PCV, pediatric patients, *S. pneumoniae*, serotype, ST1377, ST320

## Abstract

**Background:**

In Serbia, mandatory PCV immunization was introduced in 2018, following a sequential PCV10/PCV13/PCV10 schedule. This study aimed to assess changes in pneumococcal serotype distribution, vaccine serotype coverage, antimicrobial resistance (AMR), and clonal dissemination of pediatric invasive and non-invasive isolates before and after PCV introduction.

**Methods:**

A total of 626 clinical pneumococcal isolates from children were analyzed, including 251 invasive (IPD) and 375 non-invasive (NIPD) isolates. Serotyping was performed using PCR and Quellung reactions, antimicrobial susceptibility testing followed EUCAST guidelines, and invasive isolates were further characterized by multilocus sequence typing (MLST). Serotype invasiveness ratios were evaluated in pre- (2010–2017) and post-PCV (2018–2024) periods.

**Results:**

Following PCV implementation, serotype coverage declined across all vaccines, most markedly for PCV10 (IPD: 67.7–35.6%; NIPD: 62.3–17.4%), with secondary reductions in PCV13, PCV15, and PCV20 coverage largely driven by the loss of PCV10 serotypes. The proportion of non-PCV20 serotypes rose from 7.2 to 22.9% in IPD and from 12.3 to 41.6% in NIPD, with NVTs being significantly more frequent in NIPD post-PCV period. Antimicrobial non-susceptibility decreased after PCV introduction, including significant reductions in penicillin and macrolide resistance in both IPD and NIPD isolates. Resistance levels remained higher among non-invasive isolates compared with invasive isolates. Multidrug resistance declined from 39.8 to 25.4% in IPD and from 61.0 to 29.5% in NIPD. MLST analysis showed broad clonal diversity, with ST473 and ST8144 predominating pre-PCV and ST1377 and ST320 post-PCV, together with the emergence of non-vaccine lineages and serotype switch in ST320 from 19F to 19A.

**Conclusion:**

Mandatory PCV immunization in Serbia has led to substantial shifts in pneumococcal serotype distribution, resembling patterns observed in countries relying predominantly on PCV10. Also, a reduction in antimicrobial resistance and an increase in genetic diversity were observed, highlighting ongoing serotype replacement and the need for continued integrated surveillance.

## 1 Introduction

*Streptococcus pneumoniae* is a leading cause of bacterial infections worldwide. Non-invasive pneumococcal diseases (NIPDs), such as acute otitis media (AOM), conjunctivitis, sinusitis, and non-bacteremic pneumonia, are prevalent, whereas invasive pneumococcal diseases (IPDs), including bacteremic pneumonia, sepsis, and meningitis, are less frequent but often severe and life-threatening.

Although IPDs are also prevalent among the elderly, they remain a leading cause of pediatric morbidity and mortality worldwide (Tan, 2012). NIPDs, particularly AOM, are common in early childhood and represent an important reservoir for transmission, serotype diversity (Pichichero et al., 2023), and antibiotic prescriptions in this age group and contribute to the growing problem of bacterial resistance (Suaya et al., 2018).

Resistance of *S. pneumoniae* to beta-lactam antibiotics and macrolides continues to represent a global challenge. In Serbia, as well as in some European countries (Romania, Spain, France), pneumococcal non-susceptibility to these first-line antibiotics exceeds 20% (European Centre for Disease Prevention and Control, 2023). In 2024, the World Health Organization (WHO) updated its Bacterial Priority Pathogens List, categorizing macrolide-resistant *Streptococcus pneumoniae* (MRSP) as a medium-priority pathogen (World Health Organization, 2024).

The prevention of pneumococcal diseases relies heavily on vaccination. To date, several pneumococcal conjugate vaccines (PCVs) are used in the pediatric population—PCV10, PCV13, PCV15, and PCV20—while PCV21 has been approved for use in adults (Kobayashi et al., 2024). These vaccines have demonstrated strong effectiveness in preventing IPD caused by vaccine-serotypes (VTs) and, to a lesser extent, non-bacteremic pneumonia and AOM (Centers for Disease Control and Prevention, 2021; World Health Organization (WHO), 2012). Additionally, PCVs effectively reduce the acquisition of nasopharyngeal colonization caused by VTs, thereby limiting transmission between individuals (Izurieta et al., 2018). However, this success has been partially offset by the phenomenon of serotype replacement, wherein non-vaccine serotypes (NVTs) have emerged as notable causes of both IPDs and NIPDs (Grant et al., 2023). Therefore, effective monitoring of pneumococcal diseases is crucial for tracking epidemiological trends and characterizing serotype distribution. Such surveillance provides essential evidence for shaping immunization strategies, guiding vaccine development, and evaluating the population-level impact of pneumococcal vaccination programs on IPDs. In Serbia, however, despite the legal obligation to report IPD (Republic of Serbia, 2016), notification rates remain low, resulting in a lack of reliable incidence data and hindering evaluation of vaccine effects on IPD rates. Nonetheless, the National Reference Laboratory (NRL) for Streptococci has conducted voluntary, laboratory-based surveillance of IPD for 15 years. Although PCV10 and PCV13 were approved in Serbia in 2009 and 2013, respectively, their use remained negligible until 2018, when they became mandatory for children under 2 years of age, with coverage reaching 90% (Institute of Public Health of Serbia, 2024).

In our previous study, we analyzed IPD serotypes in Serbia from 2010 to 2023, providing baseline data from before and during PCV10/PCV13 implementation and documenting marked serotype shifts among children under 2 years of age, the primary vaccination target group (Opavski et al., 2024). Building on these findings, the present study examines ongoing serotype dynamics in this population following the reintroduction of PCV10 into the Serbian NIP in 2024. Importantly, the serotype distribution of NIPD in Serbia has not previously been evaluated, despite its critical role as a reservoir for transmission and a source of serotype diversity. For the first time, we include pediatric non-invasive isolates alongside invasive ones, providing a more comprehensive overview of pneumococcal seroepidemiology in the post-PCV era.

This study addresses five key objectives: (*i*) to characterize serotype distribution of both IPD and NIPD among pediatric populations; (*ii*) to compare serotype distribution between the pre- and post-PCV periods; (*iii*) to assess potential coverage by higher-valent PCVs; (*iv*) to compare antimicrobial susceptibility in invasive and non-invasive pediatric isolates; and (*v*) to investigate clonal relationships among invasive pediatric serotypes.

## 2 Materials and methods

This was a nationwide, laboratory-based surveillance study primarily based on invasive *S. pneumoniae* isolates collected through continuous surveillance over a prolonged period (2010–2024). Invasive pneumococcal isolates were obtained through the NRL for streptococci, which receives isolates from clinical microbiology laboratories across Serbia for confirmation, serotyping, and further characterization. Contributing laboratories were located in three regions of Serbia: Vojvodina (6 hospitals), the City of Belgrade (7 hospitals), and Central Serbia (9 hospitals).

Non-invasive pneumococcal isolates were collected through targeted cross-sectional sampling during two study periods corresponding to the pre-PCV (2014–2016) and post-PCV (2022–2024) eras. Sampling was performed in the two largest cities, Belgrade and Novi Sad, each represented by two participating hospitals. Partial data on invasive isolates collected between 2010 and 2023 have been previously published (Opavski et al., 2024). In the present study, these data were updated with newly collected invasive isolates from 2024 and combined with non-invasive isolates to enable comparative analyses across different epidemiological periods.

IPD was defined as the isolation of *S. pneumoniae* from a normally sterile site, including blood, cerebrospinal fluid (CSF), pleural fluid, or other sterile body fluids, and encompassed clinical syndromes such as bacteremia, meningitis, and empyema. Invasive isolates were collected continuously from healthcare institutions nationwide throughout the study period. In contrast, non-invasive isolates were obtained within the framework of two structured cross-sectional surveys. Non-invasive isolates were obtained from children diagnosed with pneumonia, AOM, sinusitis, and conjunctivitis. Diagnoses of pneumonia and AOM were established according to the WHO radiologic criteria for community-acquired alveolar pneumonia and the American Academy of Pediatrics criteria for AOM diagnosis, respectively (Cherian et al., 2005; Lieberthal et al., 2013). Clinical specimens included middle ear fluid, ear swabs collected after spontaneous otorrhea, sinus aspirates, conjunctival swabs, respiratory tract samples (e.g., sputum, tracheal aspirate, bronchoalveolar lavage [BAL], and nasopharyngeal aspirates). All isolates were obtained from pediatric patients (aged ≤18 years). Isolation and identification of *S. pneumoniae* were initially performed in regional clinical microbiology laboratories using conventional microbiological methods and the VITEK® 2 automated system (bioMérieux, France). All isolates were subsequently submitted to the NRL for Streptococci for further characterization. Since 2020, MALDI-TOF mass spectrometry has been introduced and implemented in larger regional centers, as well as at the NRL, where *lytA* and *cpsA* PCR remained the confirmatory assay for all isolates (Park et al., 2010).

The *S. pneumoniae* isolates were preserved in skim-milk broth (HiMedia, India) at −80 °C. Along with the strains, demographic data (age and gender), sample type, hospital, and diagnosis were provided from regional microbiological laboratories. This research was approved by the Ethics Committee of the Faculty of Medicine, University of Belgrade (acceptance number 1322/III-11).

All invasive pneumococcal isolates were serotyped immediately upon arrival at the NRL, using the Quellung reaction, which employed the Pneumotest kit with 12 pooled antisera and specific factor sera (Statens Serum Institute, Denmark). For non-invasive isolates, serotyping was performed stepwise by multiplex PCR as previously described (Jourdain et al., 2011), with seven PCRs until a positive result was obtained. For isolates that remained negative in all PCR mixes, or when PCR results could not differentiate between serotypes, serotyping was performed using the Quellung reaction.

Antimicrobial susceptibility was assessed by the disk diffusion method for erythromycin, clindamycin, norfloxacin, tetracycline, chloramphenicol, trimethoprim-sulfamethoxazole, and vancomycin (Bio-Rad, USA), following EUCAST guidelines (EUCAST, 2025). Macrolide resistance phenotypes: constitutive MLS (cMLS), inducible MLS (iMLS), and M phenotype, were determined using the double disk diffusion method with erythromycin and clindamycin disks. Susceptibility to beta-lactam antibiotics was initially screened using oxacillin disk diffusion testing. For isolates exhibiting an inhibition zone diameter <20 mm, minimum inhibitory concentrations (MICs) for penicillin and ceftriaxone were determined using Etest and the VITEK® 2 system (BioMérieux, France). Antimicrobial susceptibility testing results were interpreted according to EUCAST standards (EUCAST, 2025). *S. pneumoniae* ATCC 49619 was used as the quality control strain. *S. pneumoniae* isolates with a penicillin MIC >0.06 mg/L and a ceftriaxone MIC >0.5 mg/L were classified as penicillin non-susceptible (PNSP) and ceftriaxone non-susceptible (CNSP), respectively (Mohanty et al., 2022). In addition, isolates categorized as I or R to fluoroquinolones and trimethoprim-sulfamethoxazole were considered non-susceptible (Lee et al., 2020).

Multidrug-resistant (MDR) *S. pneumoniae* isolates were defined as those exhibiting non-susceptibility to three or more antibiotics from different antimicrobial classes, while extensively drug-resistant (XDR) isolates were characterized by non-susceptibility to five or more antibiotics from the following seven antibiotic classes: beta-lactams, macrolides, lincosamides, fluoroquinolones, chloramphenicol, tetracyclines, and trimethoprim-sulfamethoxazole (Cillóniz et al., 2018).

A total of 149 invasive *S. pneumoniae* isolates were randomly selected from all participating hospitals, considering the distribution of serotypes, clinical importance, and resistance profiles to penicillin and macrolides. These isolates underwent multilocus sequence typing (MLST), targeting seven housekeeping genes—*aroE*, *gdh*, *gki*, *recP*, *spi*, *xpt*, and *ddl*—as described by Enright and Spratt (1998). Allelic profiles were used to assign sequence types (STs) through the PubMLST database (Jolley et al., 2018). To explore genetic relatedness among the isolates, a minimum spanning tree was constructed using the goeBURST algorithm and visualized with PHYLOViZ software (Feil et al., 2004). Clonal complexes (CCs) were defined by grouping STs sharing five or more alleles out of the seven analyzed, as previously described (Setchanova et al., 2018). Each CC was then labeled according to its predicted founder, the central ST within the group. The resulting STs were further compared with reference clones from the Pneumococcal Molecular Epidemiology Network to identify globally recognized antibiotic-resistant lineages.

Statistical analyses were conducted using SPSS version 20.0 (SPSS Inc., Chicago, IL, USA). Categorical variables were compared using Pearson’s chi-squared test or Fisher’s exact test, as appropriate. A *p*-value of < 0.05 was considered statistically significant. The invasiveness ratio (IR) was calculated for each pneumococcal serotype as the ratio between its frequency among invasive isolates and its frequency among non-invasive isolates. An IR > 1 indicates that the serotype was more often associated with IPD, whereas an IR < 1 indicates predominance in NIPD. IR was only calculated for serotypes with at least 10 isolates in total across the study period. Serotype and ST diversity were quantified using Shannon (H′) and Simpson (1 − D) indices, calculated separately for invasive and non-invasive isolates in pre- and post-PCV periods.

## 3 Results

### 3.1 Characteristics of patients

A total of 626 clinical *S. pneumoniae* isolates were obtained during the study period (Table 1). A total of 251 invasive isolates were included in the analysis. In the pre-PCV period, 133 invasive isolates originated from three regions of Serbia: Vojvodina (*N* = 26), the City of Belgrade (*N* = 88), and Central Serbia (*N* = 19). In the post-PCV period, 118 invasive isolates were analyzed, including 18 from Vojvodina, 85 from the City of Belgrade, and 15 from Central Serbia. Among invasive pneumococcal isolates included in the analysis, the median patient age was 2 years (range: 1 month–17 years). The majority of invasive isolates were obtained from children aged ≤2 years. Blood and cerebrospinal fluid were the most frequent sources of invasive isolates, accounting for 68.9 and 24.3% of cases, respectively. Sepsis was the most common invasive disease presentation. A total of 375 non-invasive isolates were included in the analysis. In the pre-PCV period, 77 isolates were collected (Belgrade, *N* = 56; Novi Sad, *N* = 21), while 298 isolates were obtained in the post-PCV period (Belgrade, *N* = 202; Novi Sad *N* = 96). In non-invasive isolates, the median patient age was 3 years (range: 1 month–14 years). The most common NIPD in both periods was AOM (55.5%), followed by pneumonia (41.3%). The largest proportion of isolates was obtained from children aged ≤5 years (79.5%). Patients with IPD were significantly younger than those with NIPD (*p* < 0.001). No statistically significant differences were observed by sex in both IPD and NIPD.

**Table 1:** Demographic characteristics of pediatric patients with invasive and non-invasive *S. pneumoniae* infection (*N* = 626) during the pre- and post-PCV periods in Serbia (2010–2024).

Characteristics	Invasive isolates		Non-invasive isolates	
	Pre-PCV *N* = 133	Post-PCV *N* = 118	Pre-PCV *N* = 77	Post-PCV *N* = 298
Age				
≤2 years	89 (66.9%)	65 (55.1%)	31 (40.3%)	117 (39.3%)
>2 to ≤5 years	23 (17.3%)	28 (23.7%)	30 (38.9%)	120 (40.3%)
>5 to ≤18 years	21 (15.7%)	25 (21.2%)	16 (20.8%)	61 (20.4%)
Sex				
Male	71 (53.4%)	59 (50%)	46 (59.7%)	170 (57%)
Female	62 (46.6%)	59 (50%)	31 (40.3%)	128 (43%)
Specimen				
Blood	84 (63.2%)	89 (75.4%)	–	–
CSF	45 (33.8%)	16 (13.6%)	–	–
Pleural aspirate	4 (3.0%)	10 (8.5%)	–	–
Joint fluid	0 (0%)	1 (0.8%)	–	–
Peritoneal fluid	0 (0%)	2 (1.7%)	–	–
BAL	–	–	0 (0%)	46 (15.4%)
Tracheal aspirate	–	–	23 (29.9%)	33 (11.1%)
Sputum	–	–	0 (0%)	8 (2.7%)
Middle ear fluid	–	–	21 (27.3%)	48 (16.2%)
Ear swab	–	–	5 (6.5%)	65 (21.8%)
Sinus aspirate	–	–	0 (0%)	7 (2.3%)
Eye swab	–	–	2 (2.6%)	12 (4%)
Nasopharyngeal swab/aspirate	–	–	26 (33.8%)	79 (26.5%)
Diagnosis				
Sepsis	80 (60.1%)	59 (50%)	–	–
Meningitis	45 (33.8%)	16 (13.6%)	–	–
Bacteremic pneumonia	8 (6.0%)	40 (33.9%)	–	–
Septic arthritis	0 (0%)	1 (0.8%)	–	–
Perithonitis	0 (0%)	2 (1.7%)	–	–
Non–bacteremic pneumonia	–	–	23 (29.9%)	123 (41.3%)
AOM	–	–	52 (67.5%)	156 (52.4%)
Conjunctivitis	–	–	2 (2.6%)	12 (4.0%)
Sinusitis	–	–	0 (0%)	7 (2.3%)

### 3.2 *Streptococcus pneumoniae* serotype distribution

**Figure 1:**
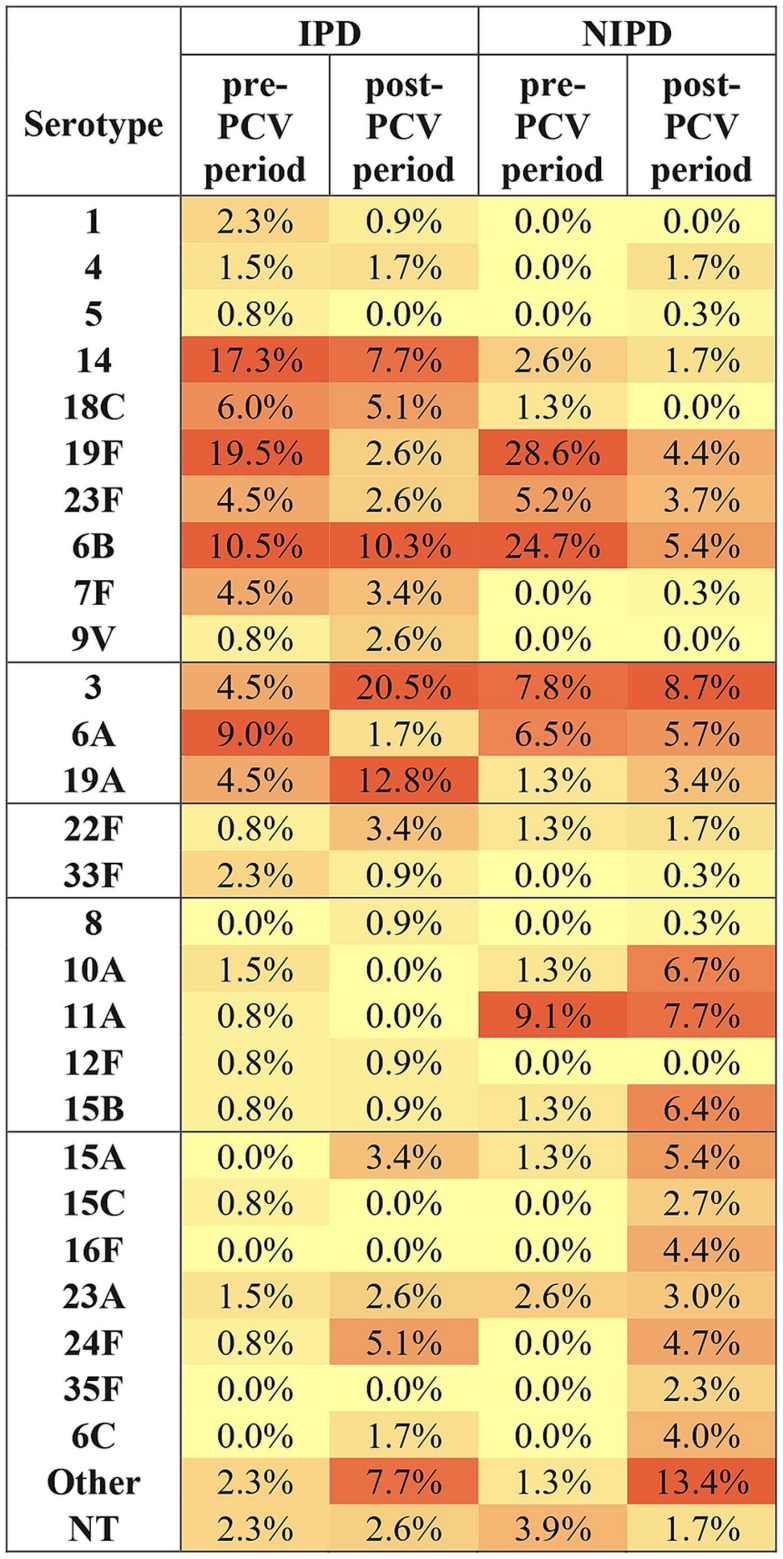
Heatmap of pneumococcal serotype distribution in invasive and non-invasive disease in the pre- and post-PCV periods. Values represent the proportion (%) of isolates within each group (IPD pre-PCV, IPD post-PCV, NIPD pre-PCV, NIPD post-PCV). Serotypes are grouped by vaccine formulation (PCV10 serotypes; additional PCV13 serotypes; additional PCV15 serotypes; additional PCV20 serotypes; non-PCV20 serotypes). NT, non-typeable; IPD, invasive pneumococcal disease; NIPD, non-invasive pneumococcal disease.

The distribution of serotypes with a prevalence greater than 1% among invasive and non-invasive infections is shown in Figure 1. Before vaccine introduction, IPD in the whole pediatric population was predominantly caused by serotypes 19F (*N* = 26; 19.5%), 14 (*N* = 23; 17.3%), and 6B (*N* = 20; 10.5%), while among NIPD, 19F (*N* = 22; 28.6%) and 6B (*N* = 19; 24.7%) were most frequent. Following PCV implementation, the overall serotype diversity increased with Shannon rising from 2.606 to 2.831 for invasive and from 2.079 to 3.308 for non-invasive isolates, and Simpson (1 − D) increasing from 0.894 to 0.909 and from 0.820 to 0.953, respectively. In IPD, the proportion of serotype 3 increased significantly from 4.5 to 20.5% (*p* < 0.001), while 19A rose from 4.5 to 12.8% (*p* = 0.023). In contrast, 19F and 14 declined from 19.5 to 2.6% (*p* < 0.001) and from 17.3 to 7.7% (*p* = 0.024), respectively. Smaller reductions were observed for 6A (9.0–1.7%), while 7F, 23F, and 18C remained stable. Among NIPD isolates, a similar decrease was recorded for 19F (28.6–4.4%) and 6B (24.7–5.4%) (*p* < 0.001 for both). Serotype distribution across different age groups is summarized in Supplementary Table 1.

Before PCV introduction, serotype 3 was most frequently associated with invasive pneumonia, while 6B was more often recovered from blood. After PCV introduction, serotype 3 increased in sepsis cases (1.5% vs. 6.8%; *p* = 0.011), whereas 19F showed a marked reduction in sepsis (12.8% vs. 1.7%, *p* < 0.001).

Otitis media as predominant NIPD was mainly associated with serotypes 6B (30.8%), 19F (26.9%), 23F (7.7%), and 6A (7.7%) in the pre-PCV period. Following vaccine introduction, the proportions of 6B and 19F declined sharply to 5.8 and 2.6%, respectively (*p* < 0.001 for both), while PCV20-nonPCV15 serotypes 10A (6.4%) and 11A (5.1%), and non-vaccine serotype 16F (7.1%) emerged in the post-PCV period. In non-invasive pneumonia, serotypes 19F (34.8%) and 6B (13.0%) declined significantly after vaccination (7.3 and 5.7%). Sinusitis and conjunctivitis were less frequent and remained associated with serotype 3.

Among the most vulnerable group, children under 5 years of age, in the post-PCV period, the most frequent serotype causing IPD was serotype 3 (*N* = 19; 20.4%), followed by 19A (*N* = 13; 14.0%), and 6B (*N* = 9; 9.7%). On the other side, among NIPD in the same age group, AOM was most commonly caused by 6B (*N* = 9; 5.8%), 11A (*N* = 8; 5.1%), 23F (*N* = 6; 3.8%), whereas non-invasive pneumonia was caused by serotype 6A and 6B (*N* = 7; 5.7% each), though these differences were not statistically significant.

The proportion of non-PCV20 serotypes among invasive isolates increased from 7.7% before PCV introduction to 23.1% in the post-PCV period, while among non-invasive isolates, NVTs rose from 9.1 to 41.6%. In the post-PCV period, NVTs were significantly more frequent in NIPD than in IPD (*p* = 0.012). In parallel, the diversity of NVTs expanded, with the number of distinct serotypes increasing from five to seven in IPD and from 8 to 16 in NIPD. In IPD, pediatric NVTs were limited to a few serotypes, primarily 23B (4.0%), 24F (3.2%), and 35B (2.8%), which together accounted for over 80% of all NVT-IPD isolates. In contrast, NIPD showed a much broader and more evenly distributed serotype profile (Supplementary Table 1).

### 3.3 Invasiveness ratio of *Streptococcus pneumoniae* serotypes

The serotype-specific IR was highest for 18C (IR = 20.9, *p* < 0.001), 14 (IR = 6.8, *p* < 0.001), and 19A (IR = 2.9, *p* = 0.005). In contrast, serotypes 11A (IR = 0.05, *p* < 0.001), 10A (IR = 0.14, *p* = 0.001), and 15B (IR = 0.15, *p* = 0.002) were significantly more common in NIPD. Several serotypes with high overall prevalence, such as 19F, 3, and 6B, showed no significant difference between invasive and non-invasive groups (Table 2).

**Table 2:** Distribution of *S. pneumoniae* serotypes in invasive and non-invasive isolates with serotype-specific invasiveness indices (IR).

Serotype	Invasive (N)	Non-invasive (N)	Total (N)	Invasive (%)	Non-invasive (%)	Invasiveness ratio (IR)	*p* value
19F	29	35	64	11.6	9.3	1.24	0.4196
3	30	31	61	12	8.3	1.45	0.1326
6B	26	35	61	10.4	9.3	1.11	0.6819
14	32	7	39	12.7	1.9	6.83	**<0.001**
6A	14	22	36	5.6	5.9	0.95	1
19A	21	11	32	8.4	2.9	2.85	**0.0047**
11A	1	30	31	0.4	8	0.05	**<0.001**
23F	9	15	24	3.6	4	0.9	0.8354
10A	2	21	23	0.8	5.6	0.14	**0.0017**
15B	2	20	22	0.8	5.3	0.15	**0.0017**
18C	14	1	15	5.6	0.3	20.92	**<0.001**

Serotypes with at least 15 isolates over the study period were included. Bold values are statistically significant significant.

Serotype 6A showed a marked reduction in invasiveness, declining from IR 2.40 pre-PCV to IR 0.12 post-PCV (*p* < 0.001). Similarly, serotype 10A, which was previously rarely detected, became almost exclusively associated with NIPD in the post-PCV period (*p* = 0.012). Serotype 19F also exhibited a significant decline in IR, shifting from 0.68 pre-PCV to 0.23 post-PCV (*p* = 0.020) (Supplementary Table 2).

### 3.4 PCV serotypes coverage

**Figure 2:**
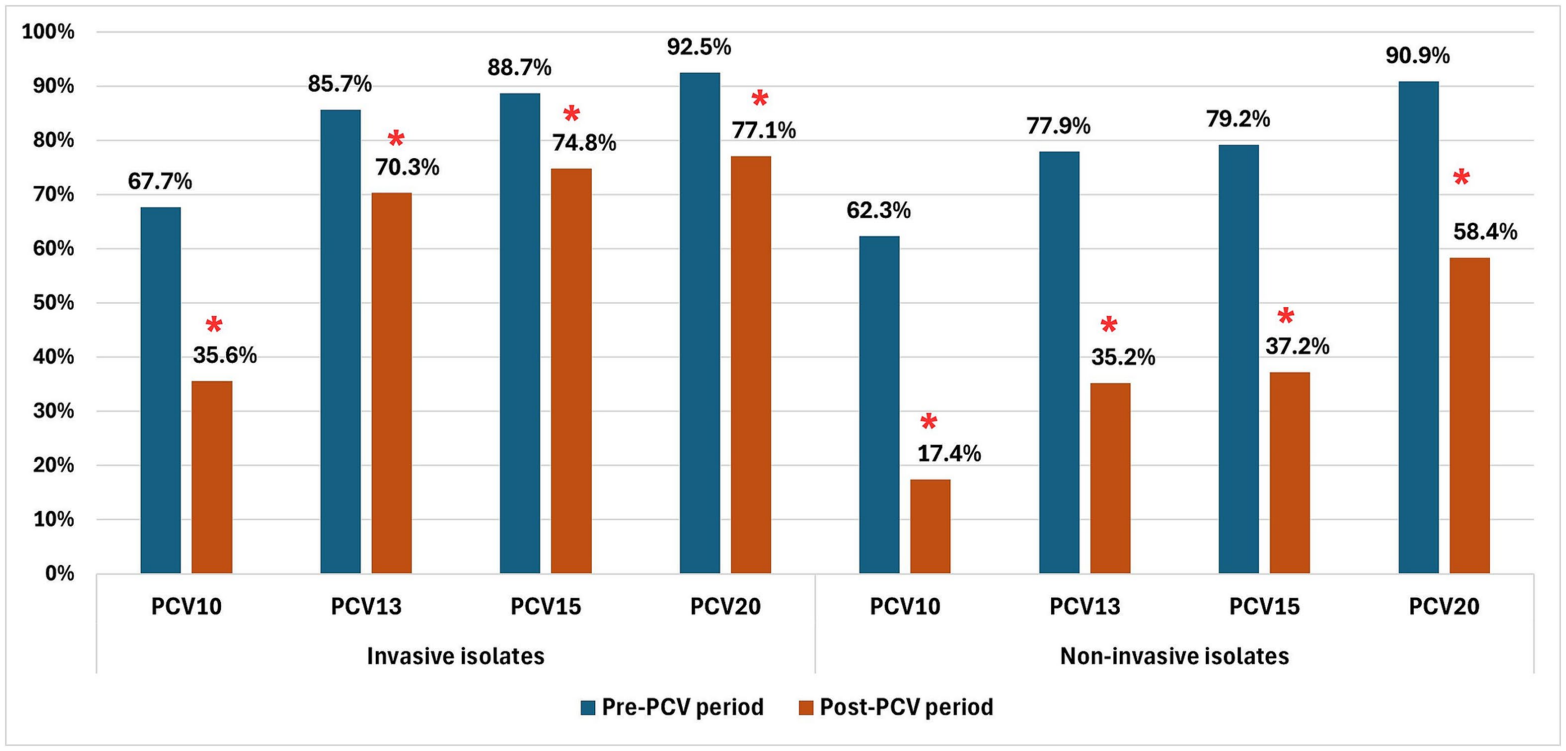
Serotype coverage of pneumococcal conjugate vaccines (PCVs) among invasive and non-invasive *Streptococcus pneumoniae* isolates before and after introduction of PCV. Bars represent the proportion of isolates whose serotypes are included in each PCV formulation (PCV10, PCV13, PCV15, and PCV20). The serotype composition of higher-valent vaccines overlaps with that of lower-valent vaccines. Asterisks indicate statistically significant differences between pre-PCV and post-PCV periods within the same PCV group (*p* < 0.05).

Overall PCV serotype coverage among invasive pediatric isolates significantly declined after vaccine introduction across all PCV formulations, with PCV10 showing the largest reduction (Figure 2). The decrease was especially marked in children younger than 2 years, dropping from 74.2 to 30.8% for PCV10 and from 87.6–92.1% to 64.6–70.8% for higher-valent PCVs (all *p* < 0.001). For NIPD isolates, coverage decreased even further (all *p* < 0.001), with PCV10 falling to just 17.4%. Among IPD isolates, the proportion of PCV13-non-PCV10 serotypes increased from 18.0 to 34.7% (*p* = 0.004). No significant changes were observed for PCV15-non-PCV13 or PCV20-non-PCV15 serotypes. By contrast, non-PCV20 serotypes expanded significantly. In NIPD isolates, PCV20-non-PCV15 serotypes increased from 11.7 to 22.1%, although the difference was not statistically significant (*p* = 0.086). Similarly to IPD isolates, the prevalence of non-PCV20 serotypes rose substantially among NIPD strains (*p* < 0.001).

**Figure 3:**
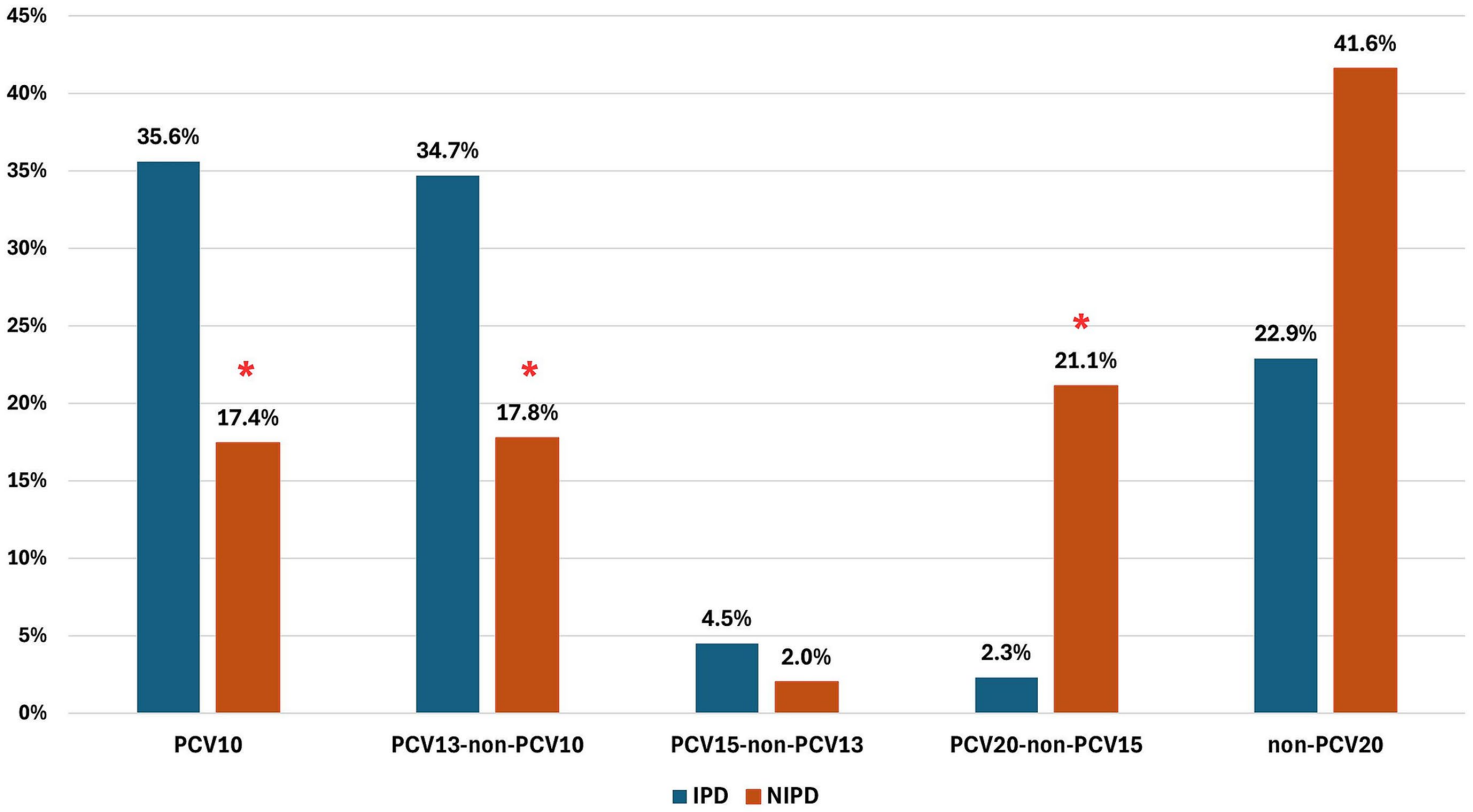
Distribution of pneumococcal serotype groups in the post-PCV period among invasive and non-invasive isolates. ^*^p < 0.05. PCV10 includes serotypes targeted by the 10-valent PCV. PCV13-non-PCV10 includes serotypes 3, 6A, and 19A. PCV15-non-PCV13 includes serotypes 22F and 33F. PCV20-non-PCV15 includes serotypes 8, 10A, 11A, 12F, and 15B. Non-PCV20 includes all other serotypes not targeted by PCV20.

In the post-PCV period, the distribution of serotype groups differed between invasive and NIPD isolates. PCV13-non-PCV10 serotypes were more common in invasive than in non-invasive isolates (34.7% vs. 17.8%, *p* = 0.005). By contrast, PCV20-non-PCV15 serotypes were markedly more frequent among non-invasive isolates (21.1% vs. 2.3%, *p* < 0.001) (Figure 3).

### 3.5 Antimicrobial resistance of *Streptococcus pneumoniae* isolates

**Figure 4:**
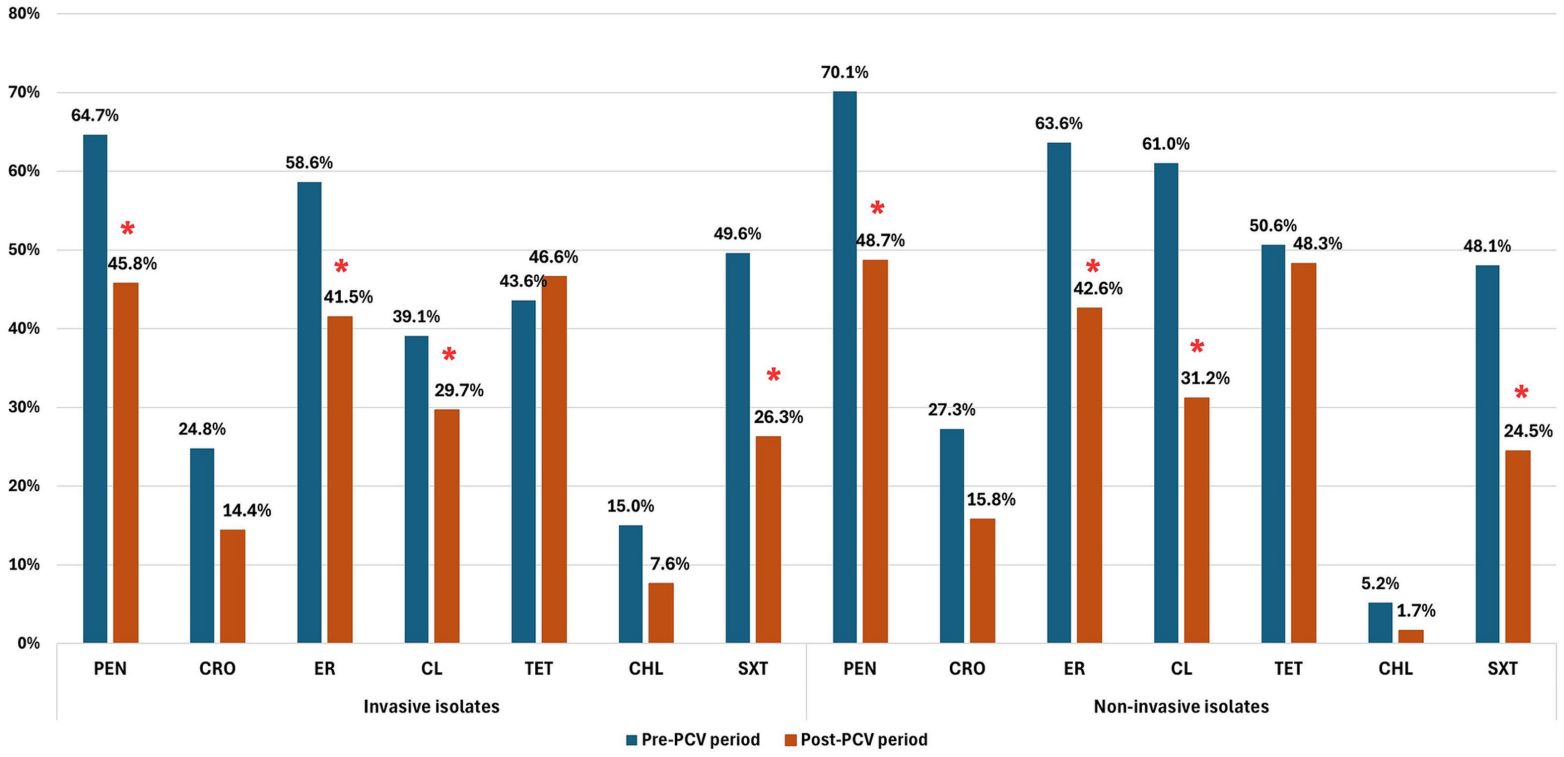
Antimicrobial non-susceptibility among invasive and non-invasive *Streptococcus pneumoniae* isolates before and after PCV introduction. ^*^p < 0.05. PEN: penicillin, CRO: ceftriaxone, ER: erythromycin, CL: clindamycin, TET: tetracycline, CHL: chloramphenicol, SXT: trimethoprim-sulfamethoxazole.

Overall, non-invasive isolates showed higher resistance rates than invasive isolates for most antibiotics (Figure 4). For penicillin, non-susceptibility declined from 64.7 to 45.8% among invasive isolates (*p* < 0.001) and from 70.1 to 48.7% among non-invasive isolates (*p* < 0.001). A similar pattern was observed for ceftriaxone, but without significant differences in both invasive and non-invasive strains. Resistance to erythromycin and clindamycin decreased significantly in both groups (both *p* < 0.001). Fluoroquinolone resistance was detected only in four invasive isolates and showed a non-significant increase post-PCV (*p* = 0.07). All isolates were susceptible to vancomycin.

In the post-PCV period, NIPD isolates remained more resistant than IPD isolates to penicillin, ceftriaxone, erythromycin, clindamycin, and tetracycline, but without statistical significance (Figure 4). In both invasive and non-invasive isolates, cMLS was the dominant phenotype of macrolide resistance, 65.2 and 75.6%, respectively. In addition, the M and iMLS phenotypes were detected, 33.3 and 1.5% in invasive, and 21.0 and 3.4% in non-invasive strains, without significant differences.

Overall, 34.8% (*N* = 218) of pneumococcal isolates were MDR, while no XDR strains were detected. MDR was more frequent among non-invasive isolates (35.9%) than in invasive isolates (33.1%), though without statistical significance. Following PCV introduction, MDR declined in both groups: among invasive isolates from 39.8% (*N* = 53) pre-PCV to 25.4% (*N* = 30) post-PCV (*p* = 0.016), and among non-invasive isolates from 61.0% (*N* = 47) to 29.5% (*N* = 88) (*p* < 0.001).

### 3.6 Sequence types of invasive *Streptococcus pneumoniae*

**Figure 5:**
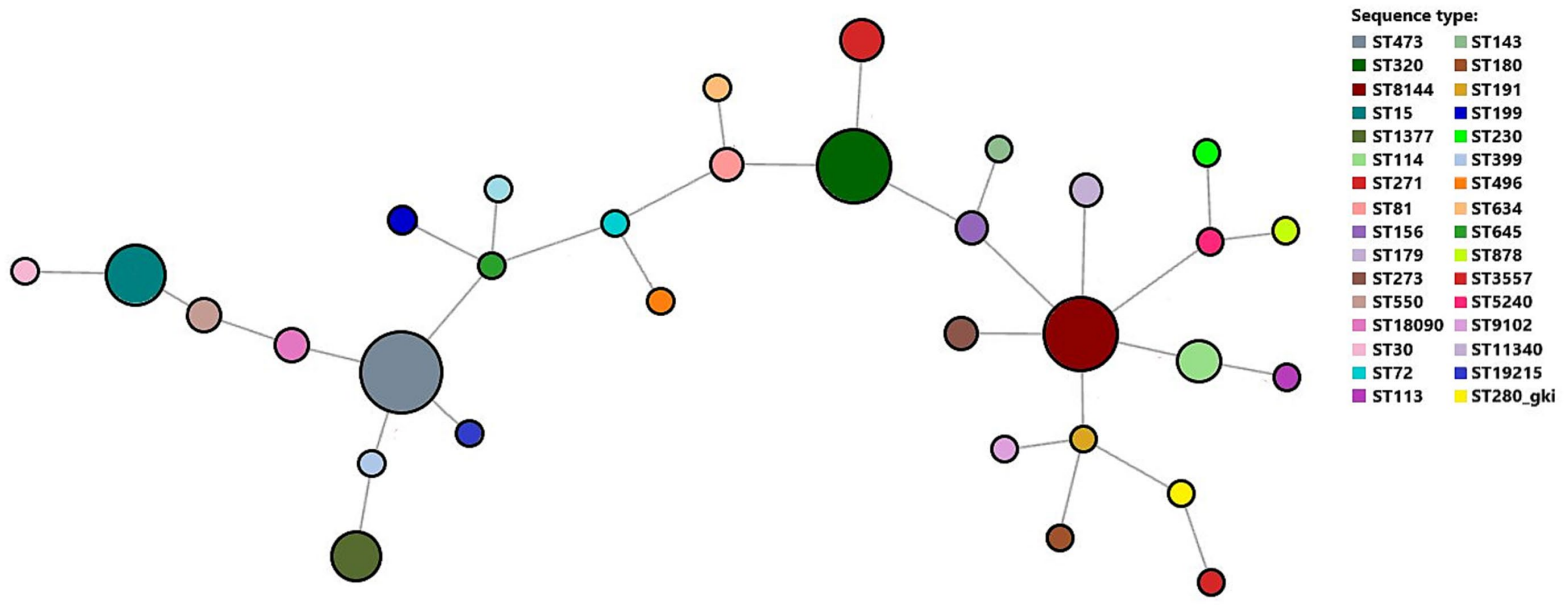
Minimum spanning tree based on the MLST scheme analysis of 68 invasive *Streptococcus pneumoniae* isolates in the pre-PCV period.

**Figure 6:**
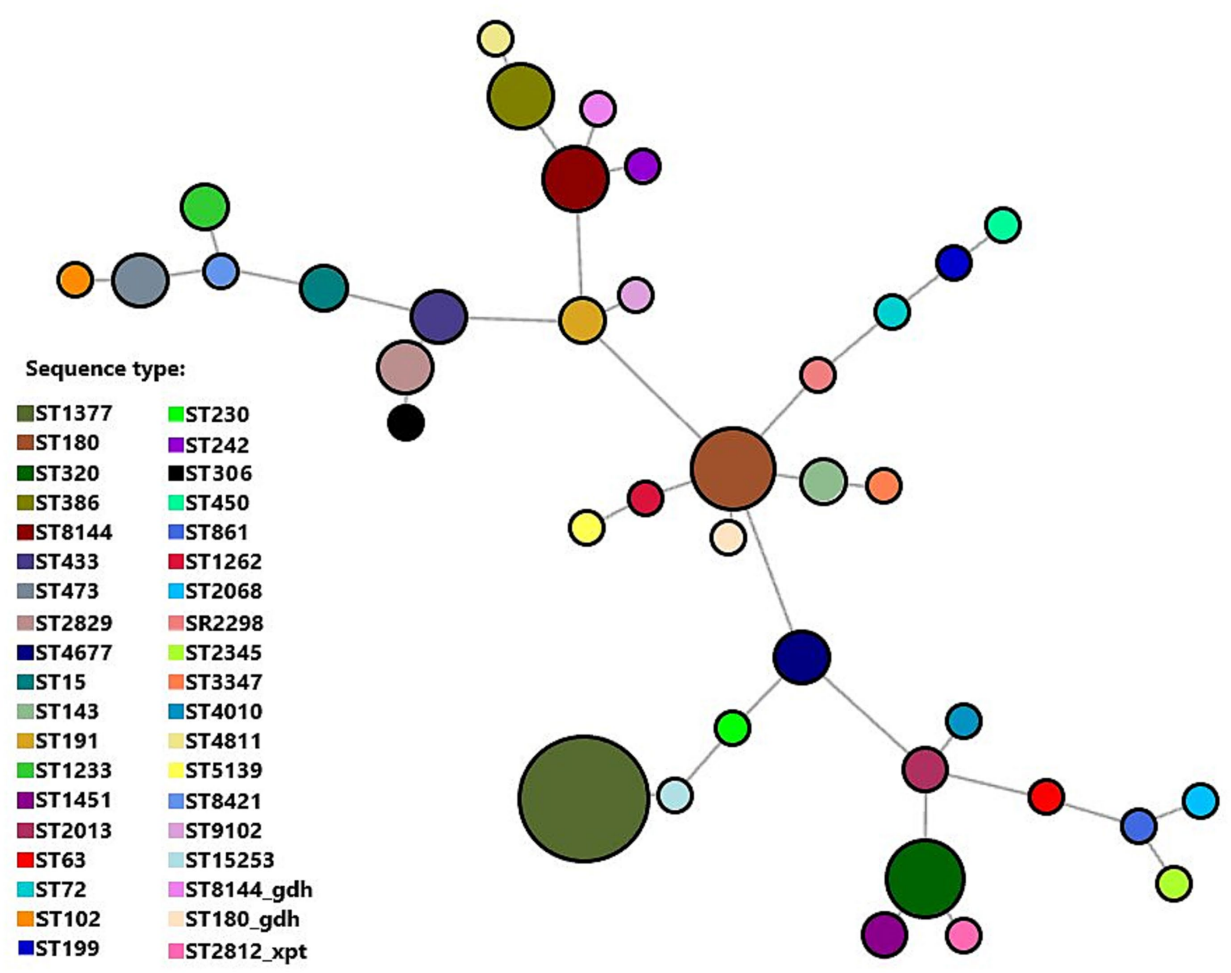
Minimum spanning tree based on the MLST scheme analysis of 81 invasive *Streptococcus pneumoniae* isolates in the post-PCV period.

A total of 149 invasive pneumococcal isolates were analyzed by MLST, comprising 68 samples collected before the introduction of PCV and 81 samples collected after its introduction. The MLST composition was broad (Supplementary Table 3). Overall, the most frequent lineages were ST1377 (*N* = 15; 10.1%), ST320 (*N* = 14; 9.4%), and ST8144 (*N* = 11; 7.4%). In the pre-PCV period, the most common STs were ST473 (*N* = 8; 11.8%), ST8144 (*N* = 7; 10.3%), and ST320 (*N* = 7; 10.3%) (Figure 5). In the post-PCV period, the most common STs were ST1377 (*N* = 11; 13.6%), ST320 (*N* = 7; 8.6%), and ST180 (*N* = 7; 8.6%) (Figure 6). Diversity indices based on ST counts indicated a modest increase post-PCV: Shannon rose from 3.155 (pre-PCV) to 3.313 (post-PCV), and Simpson (1 − D) from 0.943 to 0.949.

Several previously rare lineages, such as ST62, ST191, and ST230, emerged exclusively in the post-PCV period, often carrying non-PCV13 serotypes (10A, 15C, and 23B). A statistically significant serotype switching event was detected in ST320 (*p* = 0.004). In the pre-PCV period, it was exclusively 19F (*N* = 7; 100%), whereas in the post-PCV period, it was predominantly 19A (*N* = 6; 85.7%) (*p* = 0.004). ST1377 and ST180 were predominantly linked to serotype 3 (Supplementary Table 2). The highest MDR rates were found among ST320 and ST81, both predominantly associated with serotypes 19F and 14, respectively.

## 4 Discussion

This study is the first to compare continuously collected invasive pediatric *S. pneumoniae* isolates with non-invasive isolates obtained through two cross-sectional surveys conducted before and after PCV implementation.

The majority of patients in this study were younger than 5 years (79.9%). Bacteremia and sepsis were the most common clinical presentations of IPD, whereas AOM predominated among NIPD cases, similarly to findings in South Asia (Dhawale et al., 2025). These findings are consistent with previous studies reporting that the highest burden of both IPD and NIPD in the pediatric population occurs in children under 5 years of age, with bacteremia being the predominant clinical manifestation of IPD (Versluys et al., 2022).

Universal PCV immunization in Serbia began in 2018 after a prolonged period of limited vaccine uptake. PCV10 was introduced free of charge for children in 2018 and subsequently replaced by PCV13 in 2022. However, due to economic constraints, PCV10 was reintroduced in 2024 and is now administered according to the 2 + 1 schedule (Institute of Public Health of Serbia, 2023). Currently, two higher-valent pneumococcal conjugate vaccines, PCV15 and PCV20, are also licensed in our country. In subsequent years, alternating use of PCV10 and PCV13 created partial but sustained vaccine pressure on circulating pneumococcal populations. Considering that serotype replacement typically becomes evident three to 4 years after widespread vaccine introduction, the present findings likely reflect the early phase of post-vaccine population shifts in our setting (Izurieta et al., 2018). Despite substantial reductions in VTs (19F, 6A, and 14), serotype replacement driven by emerging non-PCV20 vaccine types (10A, 11A, and 15B) and the persistence of specific PCV13 serotypes (3 and 19A) continue to define the post-vaccination landscape. We previously conducted surveillance limited to invasive pneumococcal isolates and found that, among children under 2 years of age, PCV introduction led to a marked decline in serotypes 6B and 19F but a significant increase in serotypes 3 and 19A, which became the dominant causes of IPD in children under 5 years (Opavski et al., 2024). Also, these shifts closely parallel global trends reported in a multicenter surveillance study (Garcia Quesada et al., 2025) and Belgian national surveillance of NIPD (Passaris et al., 2024), underscoring the combined effects of direct vaccine protection and ecological replacement in the pneumococcal population.

Serotype 3 emerged as the leading cause of IPD after vaccine introduction, increasing from 4.5 to 20.5% of our pediatric isolates. Its persistence despite inclusion in PCV13 is among the most consistent global findings, often attributed to limited vaccine effectiveness against this capsule type (Zintgraff et al., 2022). The high post-PCV prevalence of serotype 3 in our results aligns with observations from Belgium (Passaris et al., 2024). Importantly, next-generation conjugate vaccines show promising potential to address this limitation: PCV15, which adds serotypes 22F and 33F, has reported higher OPA responses against serotype 3 than PCV13 (Chapman et al., 2024).

Similarly, serotype 19A remains a major contributor to pediatric IPD in the post-vaccine era. According to the global analysis, 19A accounted for approximately 30% of IPD cases among children under 5 years of age at PCV10 sites, but less than 10% at PCV13 sites (Garcia Quesada et al., 2025). Similar findings were reported in multicenter studies from Brazil (Berezin et al., 2020), Argentina (Zintgraff et al., 2022), and Mexico (Soto-Noguerón et al., 2025), where 19A persisted or expanded following the introduction of both PCV10 and PCV13.

Residual 19A carriage persists despite high PCV13 coverage, indicating that higher antibody concentrations may be required for mucosal protection than for invasive disease prevention (Grant et al., 2023). Evidence from Belgium indicates a rapid rebound of serotype 19A after the NIP reverted from PCV13 back to PCV10 (Passaris et al., 2024). The dominance of serotype 19A has been described in Bulgaria, where the PCV10 vaccine is used (Alexandrova et al., 2025). These observations suggest that switching from a higher-valent to a lower-valent PCV may compromise the serotype coverage previously achieved with PCV13.

In addition, our study indicates differences between invasive and non-invasive isolates, with higher serotype diversity observed among non-invasive isolates. This difference, characterized by broader circulation of non-PCV and PCV20-non-PCV15 serotypes in NIPD compared with a more restricted serotypes in IPD, is consistent with national surveillance data from Belgium (Passaris et al., 2024) and similar observations from Japan and Taiwan (Nakano et al., 2020; Wu et al., 2020), showing that NIPD reflects an earlier and heterogeneous stage of post-vaccine serotype replacement.

In Serbia, PCV10 coverage has remained high in recent years, exceeding 90% in the first year of life, although coverage in the second year has been consistently lower (Institute of Public Health of Serbia, 2024). Despite this, persistence of serotype 3 and increased frequency of 19A suggest that high vaccination coverage alone may not fully prevent expansion of residual or replacement serotypes, a pattern consistent with reports from other highly vaccinated populations (Garcia Quesada et al., 2025). Results from Europe showed that PCV13 non-10 IPD incidence increased in all vaccine policy and age groups because of an increase of serotype 3 in most groups, together with an increase in serotype 19A in PCV10, also seen in this study (Hanquet et al., 2022).

Following PCV introduction, classical vaccine-covered serotypes, particularly 14, 6A, and 19F, declined to very low levels in invasive disease, reflecting sustained vaccine-driven herd effects (Grant et al., 2023). Parallel expansion of non-PCV13 serotypes such as 10A, 11A, 15B, 16F, and 23B mirrors the serotype replacement reported across Europe and the United Kingdom (Levy et al., 2019; Devine et al., 2017). Similar transitions were observed in Japan (Nakano et al., 2020), Korea (Park et al., 2019), and Taiwan (Wu et al., 2020), where surveillance studies likewise demonstrated expansion of non-PCV13 serotypes and increased heterogeneity of pneumococcal populations. Notably, all of these serotypes, except for 15B, are included in PCV21, a newly approved 21-valent pneumococcal conjugate vaccine authorized by the European Medicines Agency for use in adults aged 18 years and older (European Medicines Agency, 2024). This may be of future relevance for pediatric immunization strategies, given the increasing prevalence of these serotypes. Notably, serotype 8, one of the leading invasive serotypes in several European Union countries, was not detected in our study (European Centre for Disease Prevention and Control, 2023).

In this study, serotype 3 was mainly associated with invasive pneumonia, whereas serotype 6B was more frequently isolated from bacteremia. Despite its predominance among invasive isolates, serotype 3 exhibited a low invasiveness ratio, consistent with global analyses, suggesting that its disease burden is driven more by prolonged carriage and limited vaccine-mediated clearance than by high intrinsic invasiveness (Garcia Quesada et al., 2025; Brueggemann et al., 2021).

Invasiveness ratios in this study were consistent with international patterns, with serotypes 18C, 14, and 19A remaining among the most invasive, whereas non-PCV13 serotypes such as 10A, 11A, and 15B exhibited markedly low invasiveness. This gradient in invasive potential parallels the approximately 50-fold variation observed between the most and least invasive serotypes in the population-based study by Yildirim et al. (2010) where serotypes 18C, 33F, 7F, 19A, 3, and 22F had the highest invasive capacities, while 6C, 23A, 35F, 11A, 35B, 19F, 15A, and 15B were among the lowest. Such wide variation underscores the biological heterogeneity of pneumococcal serotypes and supports the concept that newly emerging non-vaccine types typically possess lower intrinsic invasiveness (Garcia Quesada et al., 2025; Brueggemann et al., 2021).

Given the changes in serotype distribution, a decline in coverage by lower-valency PCVs is expected. In our pediatric population, PCV10 serotype coverage among invasive isolates declined from 67.7 to 35.6%, while PCV13 coverage dropped from 85.7% to 70.3%. Similar reductions in VTs coverage have been reported across Europe and Asia (Hanquet et al., 2022) and long-term surveillance in Taiwan (Wu et al., 2020), reflecting the global effect of serotype replacement and diversification of NVTs. Among children under 2 years of age, coverage with higher-valency PCVs showed a marked decline compared to earlier formulations, confirming efficient elimination of vaccine-targeted serotypes but rapid replacement by non-PCV20 types. Furthermore, European Centre for Disease Prevention and Control surveillance data show a progressive reduction in the proportion of pediatric invasive pneumococcal disease attributable to PCV10 and PCV13 serotypes between 2018 and 2022, accompanied by a growing contribution of non-PCV serotypes (European Centre for Disease Prevention and Control, 2023).

Resistance to penicillin, erythromycin, and clindamycin declined significantly in both invasive and non-invasive isolates, consistent with the disappearance of known resistant vaccine serotypes (19F, 6B, and 14). In our study, the prevalence of MDR decreased significantly following PCV introduction, from 39.8 to 25.4% among invasive isolates and from 61.0 to 29.5% among non-invasive isolates. Sustained declines in beta-lactam and macrolide resistance have been observed in the United Kingdom and Ireland following two decades of conjugate vaccine use (Horner et al., 2025), in Lebanon, where MDR halved after PCV13 introduction (El Zein et al., 2025), and in Colombia, where post-PCV10 surveillance of acute otitis media isolates showed a similar reduction in MDR (Coronell-Rodriguez et al., 2025). Comparable regional trends were reported from Korea and Taiwan, both demonstrating sharp declines in beta-lactam and macrolide resistance following vaccine implementation (Wu et al., 2020; Park et al., 2019).

Despite these encouraging trends, moderate levels of non-susceptibility to beta-lactams and macrolides persist, particularly among non-invasive isolates. Similar post-PCV resistance profiles have been described in several countries, including Greece and Japan, where overall resistance declined but did not disappear (Nakano et al., 2020; Maraki et al., 2024). By contrast, in China, macrolide resistance rates remain among the highest globally (>90%), dominated by 19F, 19A, and 14 isolates (Fu et al., 2019).

Molecular typing revealed a moderately diverse population structure among invasive isolates, with clonal diversity slightly increasing after vaccine introduction. The post-PCV period was characterized by a transition from classical vaccine-associated clones (ST320/19F, ST81/14, ST473/6B) toward serotype 3-linked lineages (ST1377 and ST180) and newly emerging non-vaccine sequence types such as ST62, ST191, ST230, and ST199, associated with serotypes 10A, 15C, and 23B. These changes closely parallel post-vaccination diversification reported in Belgium, Korea, and Taiwan, where expansion of non-vaccine lineages such as ST63/15A, ST199/15B, ST433/22F, and ST558/35B accompanied declines in vaccine-type clones (Passaris et al., 2024; Wu et al., 2020; Park et al., 2019; Gladstone et al., 2019; Lo et al., 2019). A significant capsule-switching event was detected within ST320, which expressed serotype 19F exclusively in the pre-PCV period and predominantly 19A post-PCV. This observation aligns with the adaptive plasticity of clonal complex 271 (CC271), a globally disseminated MDR lineage (Brueggemann et al. 2021; Fu et al., 2019; Croucher et al., 2015). Although the MDR genotype characteristic of the CC271 lineage likely persisted within ST320, the proportion of MDR isolates declined significantly, suggesting a gradual replacement by less-resistant descendants, consistent with observations from South Korea (Park et al., 2019).

In contrast, ST1377 and ST180, both associated with serotype 3, exhibited low resistance and stable prevalence, confirming the global persistence of this lineage despite limited vaccine effectiveness (Grant et al., 2023; Nakano et al., 2020). The emergence of ST230/10A and ST191/15C further illustrates the early diversification of non-vaccine lineages in Serbia, a pattern already observed globally (Passaris et al., 2024; Gladstone et al., 2019; Lo et al., 2019). Large genomic analyses support these findings, showing that global post-PCV diversification is driven by the expansion of pre-existing lineages rather than by *de novo* clones, particularly within CC63, CC180, and CC271 complexes (Gladstone et al., 2019; Lo et al., 2019). Together, these data indicate that clonal replacement in Serbia is in its early phase, likely reflecting the relatively short period since PCV implementation.

This study has several limitations: (i) invasive isolates were collected through laboratory-based surveillance activities, which rely on passive and voluntary submission of isolates from participating microbiology laboratories to the NRL as part of routine diagnostic and surveillance practice; (ii) the limited number of non-invasive isolates; (iii) non-invasive isolates were collected through targeted cross-sectional sampling during two defined study periods rather than continuous surveillance, and differences in sample size and specimen composition between the study periods may partly reflect variations in sampling practices and participating sites; (iv) genotyping was performed only on a representative subset of invasive isolates due to financial constraints; and (v) the absence of individual vaccination status data. Nevertheless, this study, for the first time, provides a comprehensive assessment of the pediatric *S. pneumoniae* population over a 15-year period, including their evolution, changes, and circulation.

## 5 Conclusion

Following the introduction of mandatory PCV immunization in Serbia, substantial changes in pneumococcal population structure were observed in children, with shifts in serotype distribution in both invasive and non-invasive disease. Antimicrobial resistance decreased significantly, particularly for penicillin non-susceptible isolates, with a marked reduction in multidrug-resistant strains. As expected, increased genetic diversity was observed, reflecting the expansion of clonal lineages associated with emerging NVTs in the post-vaccine period. Higher-valent vaccines such as PCV15 and PCV20 could potentially may further improve serotype coverage in the current epidemiological setting. Importantly, these changes underscore that pneumococcal epidemiology is highly context-dependent, varying according to the vaccine formulation in use, population coverage, baseline serotype distribution, and local antimicrobial resistance patterns. Therefore, continuous, country-specific, and integrated surveillance of invasive and non-invasive pneumococcal disease remains essential to inform vaccine policy decisions and optimize prevention strategies.

## Data Availability

The raw data supporting the conclusions of this article will be made available by the authors, without undue reservation.
